# Clearing of Foreign Episomal DNA from Human Cells by CRISPRa-Mediated Activation of Cytidine Deaminases

**DOI:** 10.3390/ijms21186865

**Published:** 2020-09-18

**Authors:** Sergey Brezgin, Anastasiya Kostyusheva, Natalia Ponomareva, Viktoriia Volia, Irina Goptar, Anastasiya Nikiforova, Igor Shilovskiy, Valery Smirnov, Dmitry Kostyushev, Vladimir Chulanov

**Affiliations:** 1 Department of Molecular Biology and Immunopathology of Infectious Diseases, National Medical Research Center for Tuberculosis and Infectious Diseases, 127994 Moscow, Russia; Seegez@mail.ru (S.B.); kostyusheva_ap@mail.ru (A.K.); ponomareva.n.i13@yandex.ru (N.P.); viktoriyavolya@yandex.ru (V.V.); vladimir@chulanov.ru (V.C.); 2 Department of Molecular Immunology, Institute of Immunology, Federal Medical Biological Agency, 115522 Moscow, Russia; ip.shilovsky@nrcii.ru (I.S.); vall@mail.mipt.ru (V.S.); 3 Izmerov Research Institute of Occupational Health, 105275 Moscow, Russia; probirka@list.ru (I.G.); utkina.anastasia@gmail.com (A.N.); 4 Department of Infectious Diseases, Sechenov First Moscow State Medical University, 119146 Moscow, Russia

**Keywords:** CRISPRa, cytidine deaminases, APOBECs, foreign DNA, deamination, innate immunity

## Abstract

Restriction of foreign DNA is a fundamental defense mechanism required for maintaining genomic stability and proper function of mammalian cells. APOBEC cytidine deaminases are crucial effector molecules involved in clearing pathogenic DNA of viruses and other microorganisms and improperly localized self-DNA (DNA leakages). Mastering the expression of APOBEC provides the crucial means both for developing novel therapeutic approaches for combating infectious and non-infectious diseases and for numerous research purposes. In this study, we report successful application of a CRISPRa approach to effectively and specifically overexpress APOBEC3A and APOBEC3B deaminases and describe their effects on episomal and integrated foreign DNA. This method increased target gene transcription by >6–50-fold in HEK293T cells. Furthermore, CRISPRa-mediated activation of APOBEC3A/APOBEC3B suppressed episomal but not integrated foreign DNA. Episomal GC-rich DNA was rapidly destabilized and destroyed by CRISPRa-induced APOBEC3A/APOBEC3B, while the remaining DNA templates harbored frequent deaminated nucleotides. To conclude, the CRISPRa approach could be readily utilized for manipulating innate immunity and investigating the effects of the key effector molecules on foreign nucleic acids.

## 1. Introduction

Intracellular sensing, recognition, and clearance of foreign DNA are fundamental defense mechanisms required for the proper functioning of human cells. Both pathogenic (e.g., genomes of microorganisms and viruses) and non-pathogenic DNAs (e.g., DNA leakages etc.) pose a threat to mammalian cells and must be cleared by innate immune responses [1,2]. Foreign DNA can be sensed by different toll-recognition receptors (TLRs) or in a TLR-independent manner [3]. Discovery of pattern recognition receptors and their role in countering viral, bacterial, and other foreign DNA was a major leap in understanding innate immunity. For the last ten years, a plethora of nucleic acid sensors has been identified [1]. A key role in recognizing intracellular DNA and responding to exogenous DNA was assigned to the signaling cascade cGAS/STING. The defensive response of cGAS/STING is based on the fact that DNA is not present in the cytoplasm under normal physiological conditions; in other words, any cytoplasmic DNA can be regarded by the cell as a signal of potential harm [4]. Additionally, DNA leaks may occur when nuclei, mitochondria, or lysosomes are damaged [5,6]. These events indicate significant damage to the cell and need to be handled accordingly. When present in the cytoplasm, DNA is rapidly bound by cGAS molecules and STING dimers are generated. These two components are the key signaling molecules in the cGAS/STING pathway. STING dimers contribute to activation of TBK-1 and IRF-3, two key factors that induce wide-spread activation of the innate immune response [7]. In addition to cGAS, a number of other factors play roles in recognizing cytosolic DNA, including absent in melanoma 2 (AIM2); a family of proteins with the pyrin and HIN (PYHIN) domain; DNA-dependent activator of IRFs (DAI); DExD/H box helicase protein 41 (DDX41); DNA-PK; and IFN-inducible protein 16 (IFI16) [1]. Together with the discovery of cytosolic DNA sensors, the existence of potential nuclear DNA sensors has been recently reported [8,9]. In contrast to cytoplasmic DNA, which almost always indicates pathology, the complete host genome normally resides in the nucleus. Thus, recognizing foreign DNA inside the nucleus is not a trivial task. Nuclear sensing of viral DNA is possible if host and viral DNA obviously differ in structure, length, or other characteristics. A number of factors have been previously described to participate in nuclear DNA sensing, including AIM2, cGAS, DAI/ZBP1, IFI16, TLR7/9, ZCCHC3, and RNA polymerase III [2]. In 2019, hnRNPA2B1 was discovered to be a major factor in the signaling cascade of nuclear DNA sensing, and its role in herpesvirus infection was described [9]. Mechanisms of cytoplasmic and nuclear foreign DNA recognition and factors involved in clearing foreign intracellular DNA are still not thoroughly elucidated. 

Previously, APOBEC proteins were shown to be foreign DNA restriction factors that can directly deaminate cytidine nucleotides in single-stranded or double-stranded DNA, resulting in C-to-T and/or G-to-A hypermutation, generation of deletions, and decay of foreign DNA [10]. The human APOBEC3 locus represents a cluster of seven genes on chromosome 22 encoding APOBEC3A (A3A), APOBEC3B (A3B), APOBEC3DE, APOBEC3C, APOBEC3F, APOBEC3G, and APOBEC3H proteins [11]. Upstream mechanisms regulating APOBEC3 protein expression are largely unknown. APOBEC3 expression can be induced by interferons (IFN) or other cytokines produced after detection of foreign DNA. The most well-characterized APOBEC3s inducers are IFN-α (implicated in transcriptional induction of A3A, A3G, and A3F) [12], IFN-γ (increases A3G and A3F mRNA levels) [13,14], and IFN-λ (induces A3A, A3B, and A3G expression) [15]. Besides IFN, NF-kB signaling plays an important role in regulating innate responses and inducing APOBEC3s [16]. Other stimuli can also regulate APOBEC3 expression directly (e.g., IL-27 [17], β-estradiol [18]) or indirectly (through TLR-ligands, chemokines, etc. [19]). Emerging data suggest that APOBEC3 expression is induced in a p53-dependent manner upon genotoxic damage and replication stress. Indeed, all APOBEC3s have been demonstrated to be transcriptionally responsive to p53 [20].

The antiviral properties of APOBEC3s have been extensively studied over the past decade in regard to retroviruses (e.g., human immunodeficiency virus 1 [HIV-1], human T-cell leukemia virus) [21], hepadnaviruses (e.g., hepatitis B virus, HBV) [12,22], flaviviruses (e.g., hepatitis C virus) [23], papillomaviruses (e.g., human papilloma virus, HPV) [24], herpesviruses (e.g., herpes simplex virus 1 and Epstein–Barr virus) [25], and others, including their deaminase-dependent and -independent activity. In particular, A3A and A3B have been shown to directly deaminate a key form of HBV in a non-hepatotoxic manner, contributing to the clearance of HBV DNA from infected cells [12], whereas restriction of HIV-1 infection by A3G is largely attributed to its deaminase-independent activities [26]. Additionally, foreign DNA clearance can be initiated in a A3A-dependent manner upon overexpression of A3A. In this pioneering study, A3A and other APOBEC3 proteins were shown to directly deaminate and destroy foreign DNA, thus representing potent foreign DNA restriction factors in human cells [27]. 

However, in the evolutionary arms race between viruses and their hosts, viruses have evolved numerous mechanisms to counteract innate immunity, impair recognition of cytosolic and nuclear sensors, prevent triggering of innate immune responses (e.g., dimerization of STAT), and directly inhibit effector antiviral proteins (e.g., inhibition of A3G by HIV-1 Vif protein). Activation of APOBEC3 proteins is largely complex and cell type-dependent, and requires an intricately regulated cascade of reactions that may contribute to successful elimination of foreign DNA as a threat to the host cell or, upon dysregulation, to genetic instability, mutations, and development of cancer. 

Overall, APOBEC3s are important effector molecules in the innate immune response that protect human cells from foreign DNA. Developing the strategies to precisely regulate APOBEC3 expression in a timely manner is important for novel antiviral drug development and fundamental studies. Here, we utilized a novel dead Cas9 (dCas9)-based approach (CRISPR activation or CRISPRa [28]) to directly transactivate A3A and A3B expression and test their effects on episomal and integrated foreign DNA. The results of the study demonstrate that APOBEC3s can be directly activated by a CRISPRa approach, resulting in substantial decay (>80%) of episomal DNA and inactivation of foreign GC-rich genes by deamination. However, integrated DNA was not affected. To conclude, exploiting CRISPRa systems for manipulating APOBEC3 expression may represent an essential step towards designing new therapeutic approaches for combating viral and microbial infections, destroying other foreign DNA hazards, and fundamental studies of cellular responses to foreign nucleic acids. 

## 2. Results

### 2.1. Design of sgRNAs for CRISPRa-Mediated Transactivation of A3A and A3B 

In order to activate transcription of target *A3A* and *A3B* genes, we utilized a previously established CRISPR-activation (CRISPRa) system based on a dCas9 protein, a nucleolytically dead Cas9 protein harboring point mutations in RuvC and HNH domains, which is fused to a p300 core, a catalytic histone acetyltransferase core domain of the E1A-associated protein p300 [29]. Recruitment of dCas9-p300 to a target gene promoter or proximal/distal enhancers by an sgRNA has been previously demonstrated to result in robust acetylation of the epigenome and activation of target gene transcription. For the majority of CRISPRa systems, potent transactivation of target gene transcription relies on multiple single gRNAs targeting regulatory gene regions [28]. The dCas9-p300 CRISPRa system provides a simplified approach due to the highly effective activation of gene transcription achieved even with a single sgRNA, as demonstrated in earlier studies [29].

**Figure 1:**
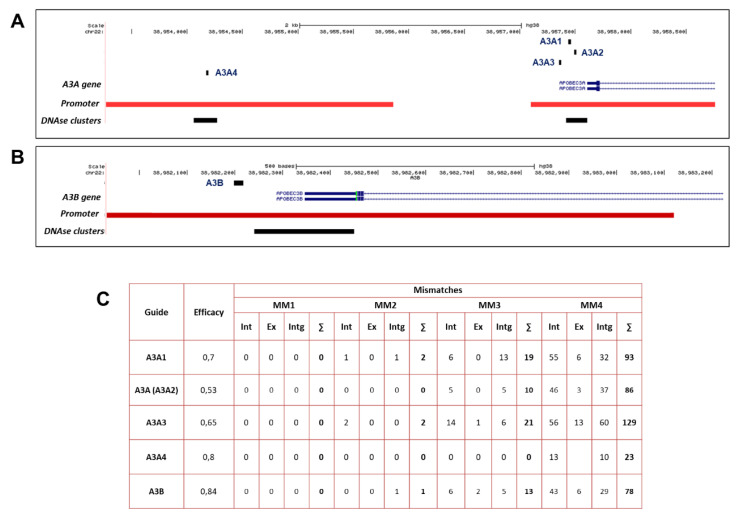
Design and characteristics of APOBEC3A/APOBEC3B-targeting sgRNAs. The (**A**) *APOBEC3A* (A3A1-4) and (**B**) *APOBEC3B* (A3B) loci on chromosome 22 shown along with sgRNA target sites, layered with ENCODE regulatory elements and DNase I sensitivity across cell lines indicated as red and black areas, correspondingly. (**C**) Characteristics (efficacy and off-target effects) of sgRNAs targeting *A3A* (A3A1-A3A4) and *A3B* promoters. Efficacy is predicted based on CCTop Broad Institute Online Calculator. MM1–MM4 stand for the number of off-target sites in introns (Int), exons (Ex), and intergenic regions (Intg) (∑ = total number of off-target sites) with the designated number of mismatches (MM1–MM4).

*A3A* and *A3B* genes lie in a tandem gene cluster on chromosome 22 together with the rest of the APOBEC3 genes. First, we designed a set of sgRNAs targeting *A3A* and *A3B* promoters. sgRNAs were designed using CCTop CRISPR/Cas9 target online predictor and UCSC genome browser [30]. sgRNAs were designed considering DNase I sensitivity, presence of DNase clusters, and position relative to transcription start site (TSS) (Figure 1A,B), as first reported by Hilton et al. [29]. DNase I sensitivity sites serve as docking spots for transcriptional and chromatin modifiers, modulating gene expression [31]. The *A3A*- and *A3B* sgRNA-targeting sites are shown in Figure 1. SpyCas9 is well known to have low mismatch tolerance and binds to DNA sites that differ by only up to 4–5 nucleotides from the sgRNAs [32]. Among the sgRNAs with suitable characteristics, we selected those with the minimal number of predicted off-target sites considering DNA:sgRNA mismatches of up to 4 nucleotides. The number of predicted off-target sites for each of *A3A*- and *A3B*-targeting sgRNAs is provided in Figure 1C. As efficacy of A3A sgRNA was relatively low (see below), we considered additional sgRNAs targeting the *A3A* promoter with high predicted scores (Figure 1C). 

### 2.2. Assessment of Single sgRNAs Targeting A3A and A3B Promoters

**Figure 2:**
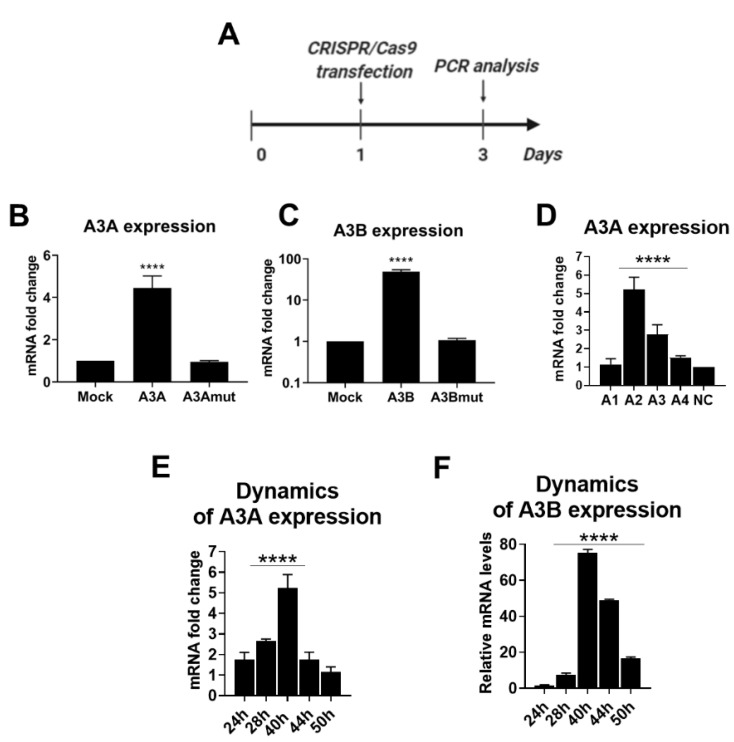
Transactivation of *A3A* and *A3B* by CRISPRa. (**A**) Experimental design. CRISPRa system was transfected into HEK-293T cells the day after seeding. APOBEC mRNA levels were measured 2 days post transfection. (**B**) *A3A* and (**C**) *A3B* mRNA expression levels upon CRISPRa compared to dCas9p300+NC sgRNA (Mock) or dCas9p300mut+NC sgRNA (mut). (**D**) Elevation of *A3A* transcription using CRISPRa with different sgRNAs (A3A1-A3A4). Dynamic analysis of (**E**) *A3A* and (**F**) *A3B* mRNA levels upon CRISPRa. *A3A/A3B* mRNA was normalized to GAPDH mRNA. Asterisks indicate statistically significant differences in means. **** *p* < 0.0001. Mock: dCas9-p300 expressing a plasmid with a non-targeting sgRNA; A3A/A3Bmut: dCas9-p300mut expressing plasmid with the corresponding sgRNA.

We co-transfected dCas9-p300 with each of the individual sgRNAs and analyzed A3A and A3B levels on day 2 post transfection (Figure 2A). As controls, we co-transfected either dCas9-p300 expressing plasmid with a non-targeting mock control sgRNA (an sgRNA that does not have targets in the human genome) or a plasmid expressing dCas9-p300 with a D1399Y mutation in the acetyltransferase domain, which abolishes its acetyltransferase activity (dCas9-p300mut) together with a corresponding *A3A*- or *A3B*-targeting sgRNA. Targeting dCas9-p300 with sgRNAs to *A3A* and *A3B* significantly elevated downstream *A3A* and *A3B* expression by up to 4- and 50-fold, respectively, compared to mock controls (Figure 2B,C). In contrast, dCas9-p300mut did not affect APOBEC mRNA-expression levels compared to mock controls. As elevation of *A3A* transcription was relatively low upon CRISPRa, we tested additional sgRNAs targeting the *A3A* promoter with high predicted scores (A3A1, A3A3, and A3A4) (Figure 1A,C and Figure 2D). sgA3A3 and A3A4 also increased A3A mRNA levels, but less profoundly compared to A3A (A3A2). sgRNA A3A1 did not have an effect on *A3A* mRNA levels. Thus, we used A3A (A3A2) in further experiments. 

An important parameter of the CRISPRa approach is the duration of target gene activation. To understand how long the activation of *A3A* and *A3B* transcription is maintained, we analyzed A3A and A3B levels dynamically (Figure 2E,F). In co-transfection experiments, *A3A* and *A3B* activation was transient, reaching the peak by 40 h post transfection and then subsiding to near-baseline levels 50 h post transfection. 

### 2.3. CRISPRa-Mediated A3A and A3B Overexpression Inhibits Transient Gene Expression

**Figure 3:**
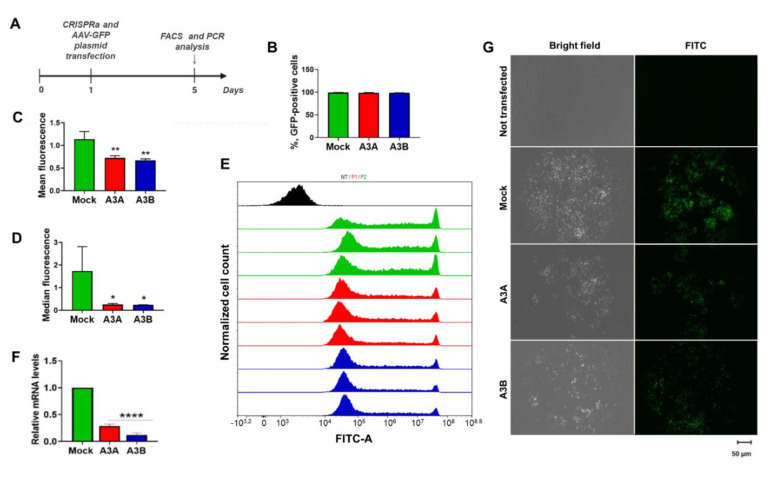
CRISPRa transactivation of *A3A* and *A3B* decreases GFP signal from an episomal plasmid. (**A**) Experimental design. HEK-293T cells were co-transfected with a GFP-expressing plasmid and CRISPRa targeting either *A3A* or *A3B* and analyzed 5 days post transfection. (**B**) Bar graph representing percentage of GFP-positive cells in experimental groups. (**C**) Mean and (**D**) median fluorescence intensity of transfected cells. (**E**) Representative FACS plots of untransfected cells (black histogram) and GFP-expressing cells co-transfected with CRISPRa and non-targeting sgRNA (green histogram), *A3A*-targeting sgRNA (red histogram), or *A3B*-targeting sgRNA (blue histogram). (**F**) Semi-quantitative RT-PCR analysis of GFP RNA upon CRISPRa of *A3A* and *A3B* genes. GFP mRNA levels were normalized to GAPDH mRNA. (**G**) Fluorescent images of untransfected, mock-transfected, and CRISPRa-transfected cells. Asterisks indicate statistically significant differences in means. * *p* < 0.05, ** *p* < 0.01, **** *p* < 0.0001.

Certain APOBECs have long been known to be involved in antiviral defense, ingestion of pathogens, and response to leaked intracellular DNA, as well as in clearance of foreign DNA. APOBECs recognize GC-rich motifs in foreign DNA and deaminate nucleotides, resulting in C-to-T and G-to-A mutations, and ultimately, degradation or mutational inactivation of the transgene [27]. To test if CRISPRa-mediated transactivation of *A3A* and *A3B* impairs stability and integrity of foreign DNA, we co-transfected CRISPRa systems targeting either *A3A* or *A3B* with a GFP-expressing plasmid (GC-rich DNA; Figure S1) and analyzed GFP fluorescence on the 5th day post transfection using flow cytometry and fluorescent microscopy (Figure 3A). The 5th day was chosen to allow activated APOBECs to affect the GFP-expressing plasmid. Transactivation of *A3A* and *A3B* did not change the percentage of GFP-positive cells (Figure 3B), but it substantially decreased GFP fluorescence intensity compared to a mock-treated control, as indicated by FACS (Figure 3C–E), PCR analysis of GFP RNA (Figure 3F), and fluorescent microscopy (Figure 3G). Indeed, analysis of cell distribution by GFP fluorescence demonstrated a decrease in cells with high GFP signal and an increase in cells with lower levels of GFP expression (Figure S2).

**Figure 4:**
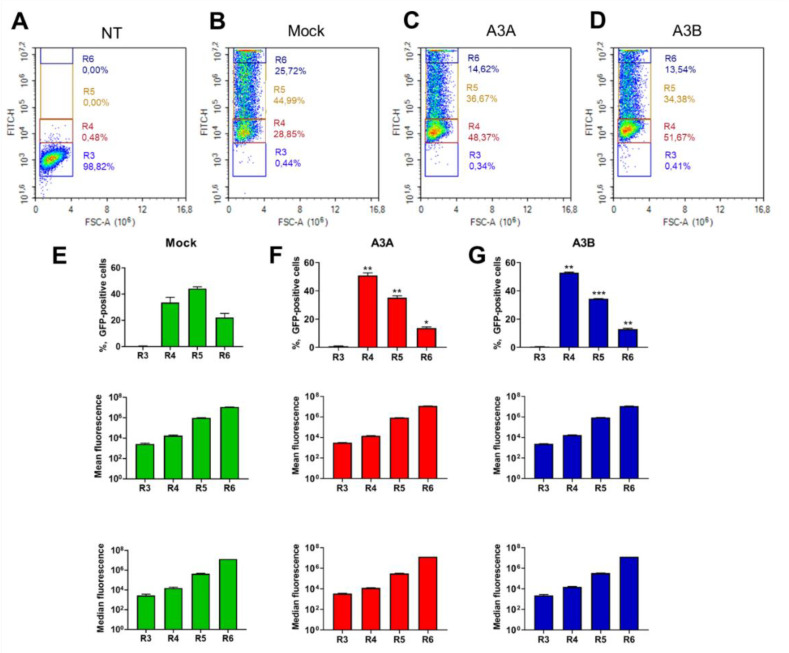
Analysis of episomal GFP fluorescence in CRISPRa-transfected cells. Histograms of GFP fluorescence in low-signal (R3) to high-signal (R6) populations of (**A**) untransfected (NT), (**B**) mock-transfected, (**C**) A3A-induced, and (**D**) A3B-induced groups. Semi-quantitative analysis of the % of GFP-positive cells and mean and median GFP fluorescence in (**E**) mock-treated, (**F**) A3A-induced, and (**G**) A3B-induced groups. * *p* < 0.05, ** *p* < 0.01, *** *p* < 0.001.

Further analysis of GFP-expressing cell populations (Figure 4) revealed a substantial reduction in populations with very high (R6; 22.2% ± 3.11% in the mock group, 13.67% ± 0.83% in A3A group, and 12.86% ± 0.62% in A3B group) and intermediate (R5; 44.24% ± 1.27% in mock, 35.16% ± 1.31% in A3A, and 34.45% ± 0.1% in A3B) GFP expression levels and a corresponding increase in low-fluorescence R4 population (33.16% ± 3.74% in mock, 50.47% ± 1.83% in A3A, and 52.22% ± 0.57% in A3B). Still, CRISPRa transactivation of *A3A* and *A3B* did not clear GFP from even a low proportion of cells, as the percentage of GFP-null cells changed only 0.3%–0.4% in the various groups (Figure 4). The rate of GFP fluorescence decay was similar between A3A and A3B CRISPRa groups, and slightly, but not statistically significantly, higher in the A3B group. This corresponds with a higher decline in GFP RNA levels upon *A3B* activation compared to *A3A* (Figure 3F; *p* < 0.01).

### 2.4. Deamination and Degradation of Foreign DNA Using the CRISPRa Approach

**Figure 5:**
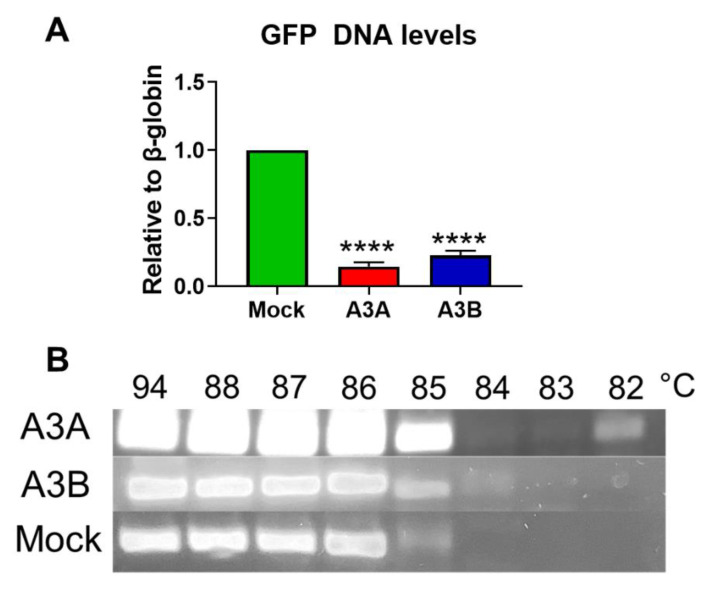
Decay and deamination of foreign DNA by CRISPRa-mediated activation of A3A and A3B. (**A**) Decline of GFP-encoding plasmid upon *A3A* (red bars) and *A3B* (blue bars) transcriptional activation by CRISPRa. **** *p* < 0.0001. (**B**) Deamination of GFP DNA by A3A and A3B measured by 3D-PCR assay.

We then asked whether the decline in GFP fluorescence was mediated by deamination of the foreign DNA, reduced DNA integrity, and, possibly, degradation of the GFP-encoding episome. Semi-quantitative PCR revealed that the amount of GFP-expressing plasmid was reduced by >86% and >78% upon *A3A* and *A3B* activation, correspondingly (Figure 5A). As has been extensively characterized in previous studies, reduced stability and degradation of the target DNA by APOBECs is induced by deamination of cytosines to produce uracil, a substrate for excision by cellular DNA repair machinery [27]. Base-pairing uridines like thymine with adenine results in typical C → T and G → A transitions. Frequent deaminated nucleotides at GC-enriched loci (like the GFP gene) are excised to produce deletions and, ultimately, extensively deaminated DNA templates are degraded. Indeed, CRISPRa-mediated activation of *A3A* and *A3B* transcription produced extensively deaminated GFP templates (Figure 5B) as evidenced by 3D-PCR. Despite the fact that by the 5th day post transfection, when DNA was isolated and analyzed, over 80% of GFP DNA in A3A and A3B groups was already destroyed (Figure 5A), we still detected amplicons amplified at temperatures as low as 82 °C for A3A group and 84 °C for A3B group (Figure 5B). 

To conclude, CRISPRa effectively induced overexpression of *A3A* and *A3B* genes, resulting in substantial decline in GFP fluorescence signal. This decline was a result of prominent destabilization and degradation of GFP-expressing plasmids. After 5 days post transfection of CRISPRa, the remaining GFP-encoding templates contained frequent deaminated nucleotides indicative of abundant deaminase activity. 

### 2.5. Short-Term Overexpression of A3A and A3B Is Not Toxic

**Figure 6:**
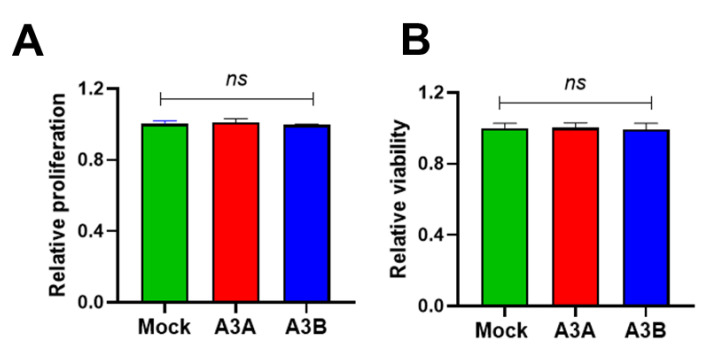
Toxicity analysis. (**A**) Cell proliferation and (**B**) viability were measured in groups transfected with CRISPRa targeting *A3A* and *A3B*. CRISPRa with a non-coding sgRNA was used as a mock control. ns: not significant.

As A3A and A3B are prominent pro-mutagenic factors that may deaminate self-DNA, introduce mutations and mutation showers, and increase genome instability and generation of highly pernicious genomic DNA double-strand breaks [33], the observed degradation of the GFP plasmid could be associated with general cellular toxicity of A3A and A3B. Thus, we transfected *A3A*- or *A3B*-targeting CRISPRa and analyzed their effects on cell viability using a commercially available cell proliferation and viability test. As demonstrated in Figure 6, CRISPRa transactivation of *A3A* and *A3B* affected neither cell proliferation nor viability 5 days post transfection compared to a mock-treated control.

### 2.6. Expression of Integrated Foreign DNA Is Not Affected by CRISPRa Transactivated A3A and A3B

**Figure 7:**
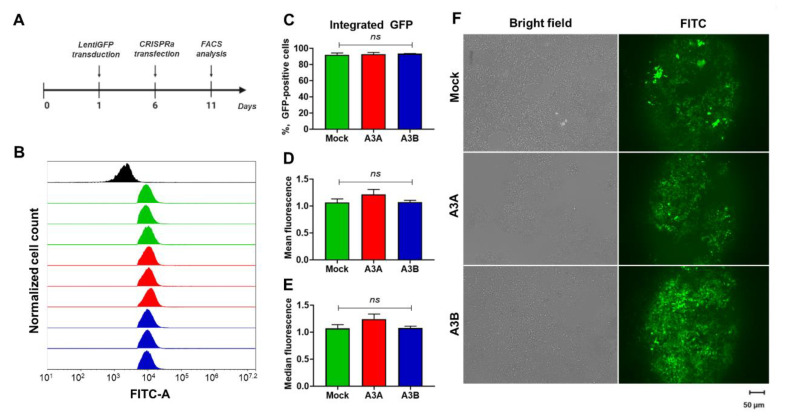
CRISPRa-activated A3A and A3B do not affect expression of integrated GFP. (**A**) Experimental design. HEK-293T cells were transduced with GFP-expressing lentivectors, transfected with CRISPRa systems 5 days later, and analyzed for GFP expression by FACS on day 11. (**B**) Representative FACS plots of not transduced (black histogram) and GFP-transduced cells co-transfected with CRISPRa and non-targeting sgRNA (green histogram), A3A-targeting sgRNA (red histogram), or A3B-targeting sgRNA (blue histogram). Bar graphs representing (**C**) percentage of GFP-positive cells, (**D**) mean and (**E**) median fluorescence intensity in experimental groups. (**F**) Fluorescent images of mock-transfected, and CRISPRa-transfected cells. ns: not significant.

Overexpression of *A3A* and *A3B* induced by CRISPRa evidently leads to decay of episomally expressed GFP. However, previous studies showed that genomic DNA may be unaffected by APOBECs [27]. To directly assess the effects of CRISPRa-induced A3A and A3B on GFP expression from an integrated construct, we transduced HEK-293T with lentiviruses carrying a GFP-expressing vector and then transfected the CRISPRa system 6 days post transfection. On day 5 post transfection, cells were harvested and analyzed by FACS and fluorescent microscopy (Figure 7). Similar to transfection experiments, lentiviral transduction of HEK-293T cells was highly effective and resulted in nearly 100% transduction efficiency. In contrast to GFP/CRISPRa-co-transfection experiments, we did not observe a decline in GFP fluorescent signal in lentivirally-transduced cells upon CRISPRa transactivation of either *A3A* or *A3B*. Indeed, the percentage of GFP-expressing cells and the mean and median fluorescence intensity were not different from mock-treated controls. Thus, we concluded that endogenously overexpressed A3A/A3B deaminases do not affect or are less effective in destabilizing integrated foreign DNA than episomally-encoded DNA. 

## 3. Discussion

Eleven APOBEC/AID proteins have been characterized to date in the human genome [34]. The majority of APOBEC/AID proteins (with the exception of APOBEC2 and APOBEC4) deaminate nucleic acids, resulting in hypermutation and generation of deletions and decay of foreign nucleic acids [34]. Most of the APOBEC/AID family members have been implicated in antiviral defense, including restriction of HIV-1 [21], HBV [12,22], HPV [24], and others. A plethora of APOBEC/AID members serves to restrict foreign DNA, such as naked plasmid DNA and DNA that leaks from mitochondria, lysosomes, or nuclei [27,35]. In particular, A3A deaminase is known to hypermutate and destroy foreign DNA transfected into human cells [27]. So far, APOBEC/AID expression in human cells is activated by regulating upstream signaling cascades mostly related to IFN and NF-kB signaling [12,13,14,15,16]. However, signal transduction pathways are usually impaired by viral proteins or dysregulated signaling that does not lead to enforced APOBEC/AID expression [36,37,38]. Another edge of this innate immunity sword is that prolonged overexpression of intracellular deaminases leads to mutagenesis of the host genome and development of cancer [33]. 

**Figure 8:**
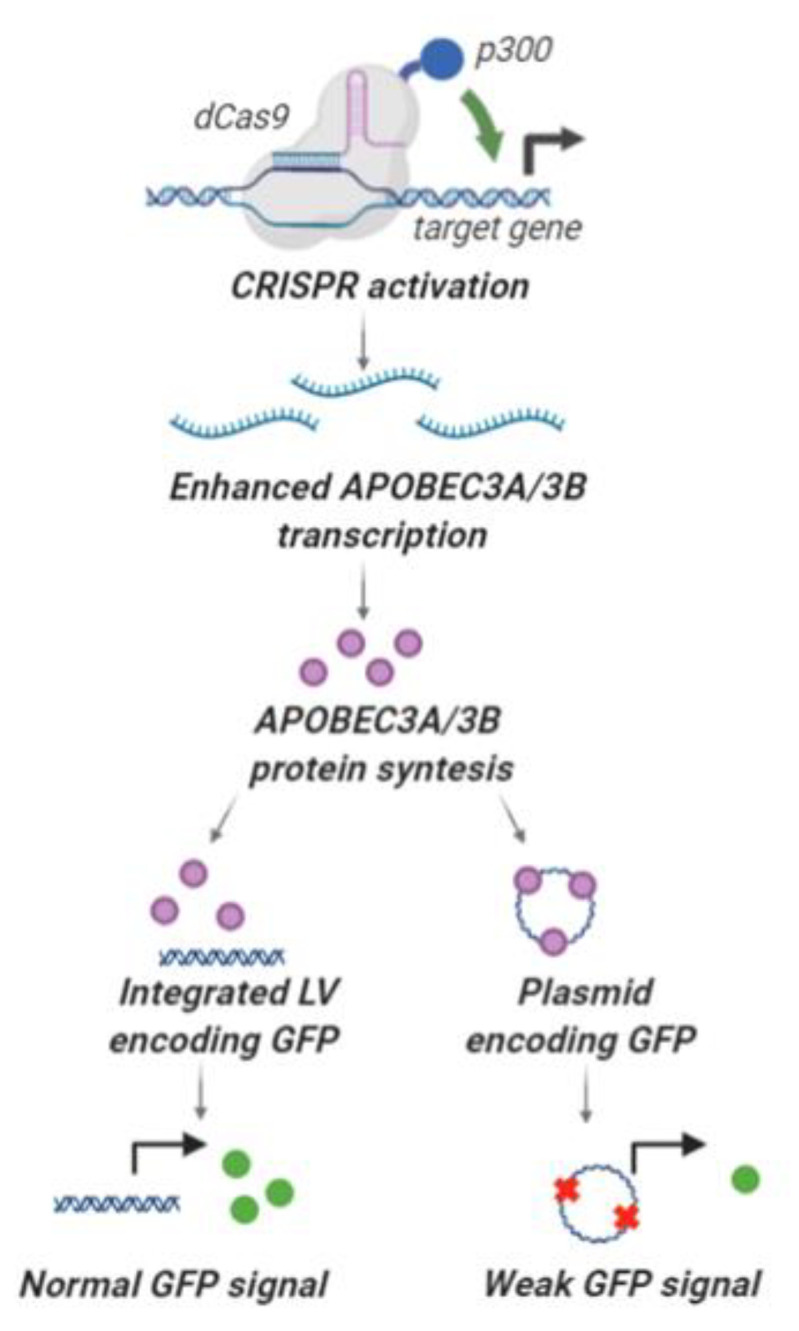
Restriction of episomally-encoded foreign DNA by A3A and A3B deaminases overexpressed by CRISPRa. CRISPRa induces specific activation of endogenous A3A and A3B deaminases that effectively suppress and destabilize episomal DNA (resulting in weak GFP signal, reduced GFP plasmid content, and mutated/deaminated GFP DNA). Integrated foreign DNA is less subjected or not subjected (at least in our experimental setting) to the disruptive effects of APOBEC cytidine deaminases. This figure was created in BioRender.

Soon after the onset of the CRISPR revolution, novel CRISPR-based platforms for transcriptional regulation were developed. In particular, novel CRISPR-interference (CRISPRi) and CRISPRa platforms were generated based on a nucleolytically null Cas9 protein. Fusing this dCas9 with a transcriptional regulator enables precise regulation of gene expression [39]. Recent methods for modulating gene expression using CRISPR-Cas systems have been reviewed elsewhere [28]. For example, Limsirichai et al. [40] and Ji et al. [41] used different CRISPRa systems to target the HIV-1 promoter in order to reactivate latent pro-virus in infected T-cells as a therapeutic strategy to clear HIV infection. As another strategy, Bogerd et al. transactivated *A3A* and *A3G* mRNA expression by CRISPRa to substantially decrease HIV-1 RNA levels [42]. Similarly, pinpoint activation of tetherin (BST-2) using CRISPRa effectively inhibited HIV-1 production and replication in vitro [43]. Besides HIV, CRISPRi/a systems have not yet been used either as an antiviral strategy or for studying the effects of innate immunity of foreign DNA. Here, we demonstrate that using a single sgRNA, CRISPRa can induce robust activation of *A3A* and *A3B*, resulting in substantial degradation of episomal DNA (Figure 8). However, integrated foreign DNA enriched in GC nucleotides (the primary targets of APOBEC3s) was not targeted by either A3A or A3B, implying that these factors are unlikely to be genotoxic for the host cells and that integrated foreign DNA sequences (e.g., integrated viral DNA or retrotransposons) will not be targeted by the cytidine deaminases. 

In this study, we used a single sgRNA targeting a single gene (*A3A* or *A3B*). Although in studies with systems other than dCas9p300 it was demonstrated that several sgRNAs demonstrate synergy and substantially increase activation of gene transcription [44], dCas9p300 was shown to robustly transactivate genes with a single sgRNA [45]. Importantly, the larger the number of different sgRNAs is introduced into human cells, the higher are the chances for off-target binding of dCas proteins and, correspondingly, for off-target activity (transactivation of undesired regulatory elements or genes) (Figure 1C). As was already observed by Hilton et al., dCas9p300 demonstrate varying efficacy on different loci. The authors previously attributed this to such factors as occupancy of genomic loci by different transcription factors, their competition, sgRNA and target loci nucleotide composition, proximity of the transcription start site, and epigenetic circuitry [29,45]. Notably, baseline expression of target genes correlates with the level of target gene activation. Indeed, *A3B* baseline mRNA was expressed <50-fold lower compared to *A3A* (Figure S2A), and was transactivated by dCas9p300 much more efficiently than *A3A*. Accordingly, the use of several sgRNA targeting *A3A* promoter did not increase *A3A* mRNA levels compared to a single, most-efficient sgRNA A3A (A3A2) (Figure S2B).

Dynamic analysis and measurement of GFP fluorescence, GFP DNA and RNA levels indicated that even a very transient upregulation of either *A3A* or *A3B* mRNA expression (Figure 2E,F) results in a substantial, but not complete, decline and deamination of foreign, episomal DNA (Figure 3, Figure 4 and Figure 5). Prolonged overexpression of APOBEC/AID by CRISPRa may be sufficient for eliminating the majority of foreign DNA, especially in such infections as chronic HBV infection. Elimination of the HBV DNA (more specifically, covalently closed circular DNA (cccDNA)—the highly persistent form of viral DNA) from infected cells is the major, final goal to cure chronic HBV infection [46]. Recently, it was demonstrated that the intracellular HBV DNA (cccDNA) pool is maintained by *de novo* infection and conversion of HBV DNA predecessor into cccDNA [47]. APOBEC/AID family members can not only destroy the major form of viral DNA [12], but also restrict de novo formation of HBV viral DNA [22,48]. Prolonged activation of APOBEC/AID factors by such approaches as CRISPRa may be sufficient to completely destroy or inactivate HBV cccDNA from infected cells and, possibly, become a cure for a chronic HBV infection. Testing CRISPRa for activating the key HBV restriction factors is instrumental for developing novel therapeutic approaches. The major challenges for the CRISPRa-based therapies include (a) the lack of effective CRISPR/Cas delivery methods; (2) the transient nature of CRISPRa activation; and (3) potential pro-mutagenic activity of APOBEC/AID factors that may contribute to mutation showers and development of cancer.

We conclude that CRISPRa-mediated activation of APOBEC3s genes can be successfully employed to neutralize the invading foreign DNA. Most importantly, the primary targets for APOBEC3s could be HBV DNA, deaminated by overexpressed *A3A* and *A3B*, and HIV-1, inhibited by overexpressed host restriction factors. Overall, the CRISPRa approach bypasses the critical steps related to inhibition of host restriction by viral proteins and directly enhances target gene transcription independently of the innate immune response. This study embodies a valuable approach for studying innate immunity and developing novel therapeutics against viral infections and genotoxicity-associated disorders.

## 4. Materials and Methods

### 4.1. Cell Culture and Transfection

Human HEK-293T (Invitrogen; Thermo Fisher Scientific, Waltham, MA, USA) cells were cultured in complete DMEM high-glucose medium with 10% FBS (Thermo Fisher), 100 U/mL penicillin, 100 µg/mL streptomycin (Sigma Aldrich, St. Louis, MO, USA), and 2 mM L-glutamine (Sigma Aldrich). The cells were seeded into 12-well plates at ~20% confluency and transfected the next day using 7.5 mM polyethylenimine. Briefly, a DNA mix containing 0.625 µg of AAV-GFP (gift from Connie Cepko; Addgene plasmid #67634, Watertown, MA, USA), 0.625 µg of pcDNA-dCas9-p300 Core or control pcDNA-dCas9-p300 Core (D1399Y) containing a mutation in p300 acetyltransferase domain (gifts from Charles Gersbach; Addgene plasmids #61358 and #61357, correspondingly), and 10 ng PCR product (with U6 promoter transcribed into a specific gRNA targeting A3A or A3B promoter) were gently added to 35 µL of NaCl solution (solution A). Solution B containing polyethylenimine (5.7 µL/well) in NaCl (29.8 µL/well) was prepared in parallel, incubated for 10 min, and gently mixed with solution A. Two solutions were incubated at ambient temperature for 10 min and then added to the cell culture medium. The day after transfection, cell culture medium was discarded, and the cells were gently washed twice in PBS and cultured in complete medium for the next 24–50 h before harvest for PCR analysis of *A3A/A3B* activation or 3–5 days for FACS analysis and GFP DNA/RNA PCR analysis (Figure 2A, Figure 3A, and Figure 7A). 

### 4.2. sgRNAs Design and Generation of sgRNA-Encoding PCR-Products Preparation

*A3A* and *A3B* promoter targets were assessed and sgRNA-designed using the open-access web tools CHOPCHOP and CCTop CRISPR/Cas9 target online calculator. Synthesis of sgRNA-encoding PCR products was performed as described in [49]. Briefly, PCR products were generated by 2-step mutagenic PCR using a high-fidelity Q5 polymerase (specific sets of primers are presented in Table 1) and purified by Qiagen gel extraction kit (Qiagen, Hilden, Germany). Concentration of synthesized PCR products was measured by Nanodrop2000. Plasmid pLX-sgRNA (Addgene #50662) was used as template for the 1-step PCR. sgRNA targets were (1) A3A1: 5′-CAAAACCAAAGCTTGCCCAG-3′; (2) A3A (A3A2): 5′-GCTAATGAGGGTGGCACACT-3′; (3) A3A3: 5′-GGCCCACAGGGAGCAAAGTG-3′; (4) A3A4: 5′-ATTCTTACCGTGAAGAGTGC-3′ (5) A3B: 5′-ATTGGAGGTTCCTCTGCCAG-3′; and (6) mock control: 5′-CTGCCTGCCTCGTCAACACC-3′.

### 4.3. Isolation of Nucleic Acids and Semi-Quantitative PCR Analysis

Cell culture medium was discarded; cells were washed twice with PBS and lysed in AmpliSens Riboprep lysis buffer (AmpliSens Biotechnologies, Moscow, Russia). RNA was isolated as described previously [50]. Briefly, nucleic acids were isolated using the AmpliSens Riboprep kit, treated with RNase-free DNase I (New England Biolabs, Ipswich, MA, USA) for 30 min at 37 °C, purified from DNase I using the AmpliSens Riboprep kit, and reverse-transcribed using AmpliSens Reverta-FL (AmpliSens Biotechnologies, Moscow, Russia). A real-time semi-quantitative PCR was performed using SYBRGreen dye (Invitrogen, Carlsbad, CA, USA). *A3A* and *A3B* mRNA levels were assessed in relation to GAPDH mRNA. Specific sets of primers and probes are presented in Table 2. GFP DNA and RNA levels were measured using real-time semi-quantitative PCR with primers listed in Table 3. GFP DNA levels were assessed in relation to β-globin. Relative expression levels were calculated via the ΔΔCt method.

### 4.4. 3D-PCR 

DNA samples were used for real-time PCR with Taq DNA polymerase, 300 nM 3D/DNA/RNA-GFP_fw/3D/DNA/RNA-GFP_rev primers (Table 3), and SYBRGreen dye. PCR conditions were as follows: 30 s at 94 °C, 30 s at 60 °C, 10 s at 72 °C (×35 cycles). Amplicons were serially diluted 1:20 to 1:50 and used for nested PCR with 300 nM 3D-GFP_fw/3D-GFP_rev primers and decreasing range of denaturation temperatures. PCR conditions were: denaturation for 60 s at 94 °C (or lower in 1 °C increments from 88 to 82 °C), 30 s at 60 °C, 20 s at 72 °C (×35 cycles). Amplicons were resolved on a 1.2% agarose gel. Alternatively, the same pair of primers was used for semi-quantitative real-time RT-PCR analysis with the following PCR conditions: 30 s at 94 °C, 25 s at 60 °C, 10 s at 72 °C (×45 cycles).

### 4.5. Production of Lentiviruses

Lentiviruses were generated as described previously [51]. In brief, HEK-293T cells were seeded into T75 TC-treated flasks at 30% initial confluency to reach ~70% confluency the next day, and co-transfected with a mix of ΔHelp plasmid (Clontech; Takara Bio, Mountain View, CA, USA), VSVg-encoding plasmid (Clontech), and lentiEGFP plasmid using polyethylenimine in NaCl. After 24 h, complete medium was replaced with OptiMem (Thermo Fisher Scientific, Waltham, MA, USA). After 48 h, conditioned medium was removed from the flasks, centrifuged at 13,400 × rpm, filtered through a 0.45-µm syringe filter (Corning Inc, Corning, NY, USA) to discard cell debris, placed in sterile 20% sucrose solution (Sigma Aldrich, St. Louis, MO, USA) in centrifuge tubes (Beckman Coulter, Brea, CA, USA), and concentrated by ultracentrifugation in SW28 rotors of a LE-80K centrifuge (Beckman Coulter, Brea, CA, USA) for 2 h at 100,000× *g* and 4 °C. The supernatant was discarded, and the concentrated lentiviruses were dried at room temperature and resuspended in complete DMEM. Aliquots were stored at −70 °C until use. 

### 4.6. Lentiviral Transduction

HEK-293T cells were plated onto 12-well plates in complete medium and allowed to adhere overnight. Lentiviruses were added to cells in complete DMEM in the presence of 8 µg/mL polybrene (Millipore, Burlington, MA, USA). After 48 h, media were discarded, and cells were washed twice in PBS and cultured in complete DMEM. 

### 4.7. Flow Cytometry and Fluorescent Microscopy

At harvest, cells were counted on a BD FACSCanto II flow cytometer (BD Biosciences, San Jose, CA, USA). Briefly, cell culture medium was discarded, and cells were washed twice in PBS, detached from the plates in trypsin-EDTA, resuspended in complete medium, and washed twice in PBS. EGFP-positive cells were detected in the FITC channel. Data were acquired with BD FACSDiva software and analyzed with NovoExpress software (ACEA Biosciences, San Diego, CA, USA). Fluorescent and bright field images were taken on an Olympus IX71 (Olympus Life Science, Tokyo, Japan). 

### 4.8. Toxicity Assays

HEK-293T cells were seeded on 96-well plates to reach ~30–40% confluence on the day of transfection. Cells were co-transfected with AAV-GFP, pcDNA-dCas9-p300 Core/control, pcDNA-dCas9-p300 Core (D1399Y), and sgRNA-encoding PCR products using polyethylenimine. Cell cytotoxicity was analyzed using Abcam Cell Cytotoxicity Assay Kit (ab112118; Abcam, Cambridge, UK) on day 5 post transfection according to manufacturer’s protocol. Optical density was measured using an iMark plate Spectrophotometer (BioRad, Hercules, CA, USA). 

### 4.9. Statistical Analyses

Values were expressed as the mean ± standard deviation (SD) of triplicate experiments in GraphPad Prism 7.0 software. Student’s T-test with Tukey’s HSD post hoc test were used to compare variables and calculate p values to determine statistically significant differences in means.

## Figures and Tables

**Table 1:** Primers for generating sgRNAs.

Primer Name	5′–3′ Sequence
sgA3A1_fw	CAAAACCAAAGCTTGCCCAGGTTTTAGAGCTAGAAATAG
sgA3A1_rev	CTGGGCAAGCTTTGGTTTTGCGGTGTTTCGTCCTTTC
sgA3A2_fw	GCTAATGAGGGTGGCACACTGTTTTAGAGCTAGAAATAG
sgA3A2_rev	AGTGTGCCACCCTCATTAGCCGGTGTTTCGTCCTTTC
sgA3A3_fw	GGCCCACAGGGAGCAAAGTGGTTTTAGAGCTAGAAATAG
sgA3A3_rev	CACTTTGCTCCCTGTGGGCCCGGTGTTTCGTCCTTTC
sgA3A4_fw	ATTCTTACCGTGAAGAGTGCGTTTTAGAGCTAGAAATAG
sgA3A4_rev	GCACTCTTCACGGTAAGAATCGGTGTTTCGTCCTTTC
sgA3B fw	ATTGGAGGTTCCTCTGCCAGGTTTTAGAGCTAGAAATAG
sgA3B rev	CTGGCAGAGGAACCTCCAATCGGTGTTTCGTCCTTTC
sgNC_fw	CTGCCTGCCTCGTCAACACCGTTTTAGAGCTAGAAATAG
sgNC_rev	GGTGTTGACGAGGCAGGCAGCGGTGTTTCGTCCTTTC
pLX-sgRNA_U6 fw	TATATAGGATCCGAGGGCCTATTTCCCATGATTCCTTCATATTTG
pLX-sgRNA_SP rev	TATATAGCTAGCAAAAAAAGCACCGACTCGG

**Table 2:** Primers and probes used for mRNA analysis.

Primer Name	5′–3′ Sequence
GAPDH fw	CAACGGATTTGGTCGTATTGG
GAPDH rev	GCAACAATATCCACTTTACCAGAGTTAA
GAPDH probe	(FAM)-CGCCTGGTCACCAGGGCTGC-(BHQ1)
β-globin fw	V31-FEP-CE-AmpliSens HPV HCR-Screen (CRIE)
β-globin rev	V31-FEP-CE-AmpliSens HPV HCR-Screen (CRIE)
β-globin probe	V31-FEP-CE-AmpliSens HPV HCR-Screen (CRIE)
A3A fw	AGATGGAGTCTGGTACTGTCG
A3A rev	GAGGCAGGAGAGTAGCGT
A3B fw	GAGCTACACTTGGCTGTGCT
A3B rev	TGACATTGGGGTGCTCAGAC

**Table 3:** Primers used for 3D/DNA/RNA-PCR.

Primer Name	5′-3′ Sequence
3D/DNA/RNA-GFP fw	AAG TTC AGC GTG TCC GGC GA
3D/DNA/RNA-GFP rev	GCG CTC CTG GAC GTA GCC TT

## Supplementary Materials

The following are available online at https://www.mdpi.com/1422-0067/21/18/6865/s1.
