# Supplementary material for: Clearing of Foreign Episomal DNA from Human Cells by CRISPRa-Mediated Activation of Cytidine Deaminases
Source: Int J Mol Sci. 2020 Sep 18;21(18):6865. doi: 10.3390/ijms21186865 (PMC7557733; doi:10.3390/ijms21186865)
Supplement: Supplementary file 1 [file ijms-21-06865-s001.pdf]

Article

Clearing of Foreign Episomal DNA from Human Cells by CRISPRa-Mediated Activation of Cytidine Deaminases

Sergey Brezgin [1,2,†], Anastasiya Kostyusheva [1,†], Natalia Ponomareva [1], Viktoriia Volia [1], Irina Goptar [3], Anastasiya Nikiforova [3], Igor Shilovskiy [2], Valery Smirnov [2], Dmitry Kostyushev [1,*,‡] and Vladimir Chulanov [1,4,‡]

1 Department of Molecular Biology and Immunopathology of Infectious Diseases, National Medical Research Center for Tuberculosis and Infectious Diseases, 127994 Moscow, Russia; Seegez@mail.ru (S.B.); kostyusheva_ap@mail.ru (A.K.); ponomareva.n.i13@yandex.ru (N.P.); viktoriyavolya@yandex.ru (V.V.); vladimir@chulanov.ru (V.C.)

2 Department of Molecular Immunology, Institute of Immunology, Federal Medical Biological Agency, 115522 Moscow, Russia; ip.shilovsky@nrcii.ru (I.S.); vall@mail.mipt.ru (V.S.)

3 Izmerov Research Institute of Occupational Health, 105275 Moscow, Russia; probirka@list.ru (I.G.); utkina.anastasia@gmail.com (A.N.)

4 Department of Infectious Diseases, Sechenov First Moscow State Medical University, 119146 Moscow, Russia

* Correspondence: dkostushev@gmail.com

† These authors contributed equally to this work.

‡ Co-senior authors.

Supplementary materials

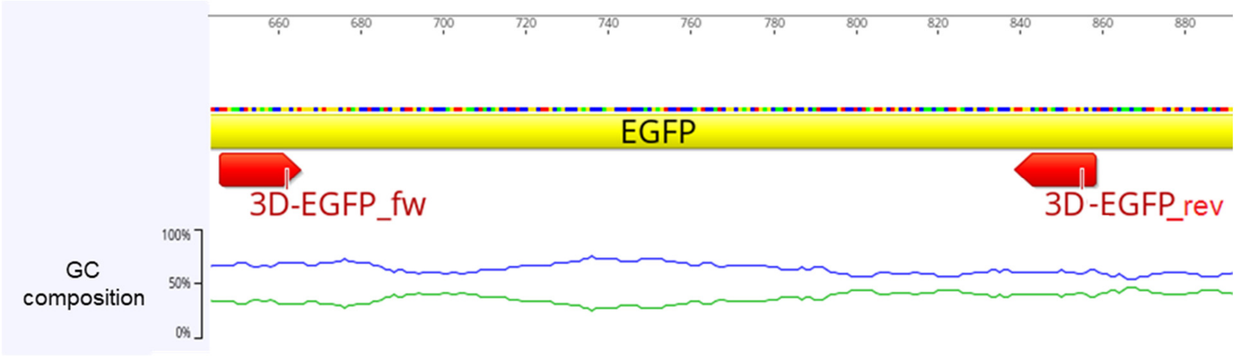

Figure S1. Schematic representation of the GFP (EGFP) locus analyzed by 3D-PCR. Blue line: GC composition; green line: AT composition. 3D-EGFP_fw/3D-EGFP_rev.

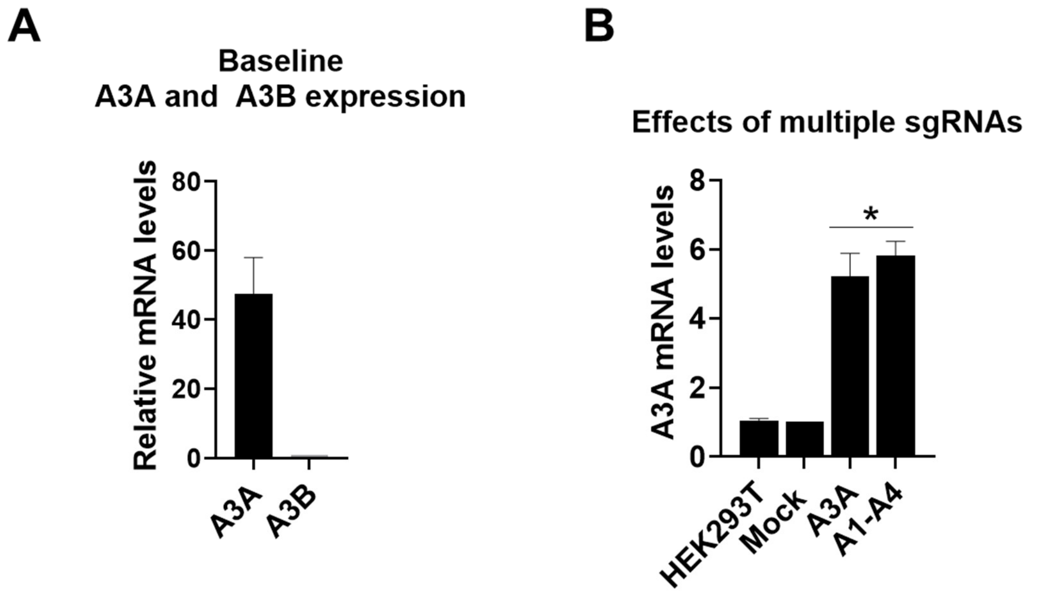

Figure S2. Baseline expression of APOBEC3A/3B and effects of multiple sgRNAs on A3A transactivation. (**A**) Baseline expression of A3A and A3B mRNAs. (**B**) Activation of A3A by a single sgRNA A3A (A3A2) or multiple sgRNAs (A3A1-4) using dCas9p300 CRISPRa system. A3A/A3B mRNA levels were normalized to GAPDH mRNA. Asterisks indicate statistically significant differences in means compared to HEK293T and Mock control. *$p < 0.0001$.
